# First Principle Study on Structural, Electronic, Magnetic, and Optical Properties of Co-Doped Middle Size Silver Clusters

**DOI:** 10.3390/molecules29112670

**Published:** 2024-06-05

**Authors:** Weiyin Li, Hao Feng, Ruiyong Shang

**Affiliations:** 1 School of Electrical and Information Engineering, North Minzu University, Yinchuan 750021, China; 17865716159@163.com (H.F.); 18790205016@163.com (R.S.); 2 Key Laboratory of Physics and Photoelectric Information Functional Materials, North Minzu University, Yinchuan 750021, China; 3 Microelectronics and Solid-State Electronics Device Research Center, North Minzu University, Yinchuan 750021, China

**Keywords:** Ag–Co cluster, optical, structure, magnetic property

## Abstract

The structural, electronic, magnetic, and optical properties of Co-doped 10–20-atom silver clusters are investigated by GGA/PBE via the density functional theory. The Ag–Co clusters form core–shell structures with a Co atom in the center. Co atom doping modulates electronic properties like energy gap, molecular softness, global hardness, electronegativity, and electrophilicity index. For the optical spectra of the Ag–Co clusters, the energy of their spectra overall exhibits little change with increasing numbers of atoms; the strongest peaks are roughly distributed at 3.5 eV, and the intensity of their spectra overall is strengthened. Raman and vibrational spectra reflect structural changes with Co atom addition. The addition of the Co atom alters magnetic moments of specific Ag–Co clusters, while others remain unchanged.

## 1. Introduction

The noble metal clusters have important applications in electronics, catalysis, optics, and medical fields because of their unique physical and chemical characteristics [1,2,3,4,5,6]. Their properties are regulated by their size, structure, and composition [7,8,9,10,11,12,13]. With the continuous development of cluster science, the study of noble metal clusters has gradually changed from pure noble metal clusters to bimetallic clusters. Bimetallic clusters exhibit many special properties that differ from pure metal clusters. However, fully revealing the properties of bimetallic clusters is very challenging due to their structures becoming complicated compared with pure metal clusters. At present, the studies show that silver–cobalt (Ag–Co) bimetallic clusters have potential applications in magnetism, optics, and catalysis [14,15,16,17,18,19,20].

Currently, the studies on Ag–Co binary clusters primarily concentrate on their structural and magnetic properties. Cheng et al. calculated the second-order difference of binding energy of a Co*_m_*Ag*_n_* (*m* + *n* = 13) cluster, indicating that the structures of the Co*_m_*Ag*_n_* (*m* = 6, 8, 10, 12) clusters are relatively stable, and studied their energy gap and magnetic properties, indicating that the Co_6_Ag_7_ cluster has high chemical stability and its magnetic property is enhanced with the increase in the number of Co atoms [21]. Dzhurakhalov et al. found that core–shell structural Ag–Co clusters form by regulating their composition and temperature [22]. Garcia-Torres et al. showed various structures of Co–Ag alloy, and special core–shell structures effectively ensure plasma loss and magnetic features [23]. Laasonen et al. concluded that Ag–Co nanoalloy clusters are all core–shell structures, with Ag atoms occupying the surface and Co atoms residing in the core, using density functional theory [24]. Ferrando et al. affirmed that silver atoms have a tendency to occupy surface positions, and that the lowest-energy clusters in the polymerized icosahedral structures of 28 and 34 atoms are intermediate components, which is attributed to the interaction of the magnetic moments of internal Co atoms in the core–shell structure, as determined by a DFT (density functional theory) study [25]. Bochicchio et al. determined the chirality of core–shell Ag–Co clusters by combining global optimization searches and first principles [26]. Kong et al. reported that Co–Ag structures can produce special magnetic moments [27]. Hoof et al. obtained the core–shell structures of Ag–Co clusters using molecular dynamics [28,29]. Janssens et al. showed that in <50-atom Co-Ag clusters, the rich Ag clusters show an obvious quantum size effect, while this effect is lacking in the rich Co clusters, and they found experimental evidence of the stability enhancement of the Ag_10_Co^+^ cluster, theoretically showing that the magnetic moment on cobalt atoms disappears [30,31]. Recently, the structural, electronic, and optical characteristics of seven- and thirteen-atom Ag–Co clusters were investigated using density functional theory by our teams [32,33].

Studying the theoretical aspects of Ag–Co clusters, including their structure, composition, and performance, holds significant importance; however, these studies are too limited to be applied. In addition, most of the research on Ag–Co clusters focuses on their structural stability, magnetic properties, and catalytic characteristics, but the electronic and optical characteristics of Ag–Co clusters are little understood. To identify novel applications in the optics fields, it is necessary to perform theoretical research on their structures and optical performances. Therefore, the structural, electronic, magnetic, and optical properties of Co-doped 10–20-atom silver clusters are studied by GGA/PBE via the density functional theory.

## 2. Model and Computational Methods

Firstly, the initial global searches of the structures of Ag–Co clusters were carried out using the artificial swarm algorithm (Artificial Bee Colony Algorithm, ABC) [34,35] with the empirical Gupta many-body potential used to model atomic interactions [36,37]. The Gupta potential parameters of Ag–Co clusters are shown in Table 1, where *r*_0_ is the atomic nearest-neighbor distance, and the parameters *A*, *ξ*, *p*, and *q* are derived from numerous experimental values of binding energy, lattice parameters, and elastic constants.

Secondly, the above initial structures of Ag–Co clusters were optimized using DFT calculations, implemented in the Dmol^3^ package included in the software Materials Studio 2019 [38,39], and the smallest structures were selected as the study objects. The optimizations and performance calculation parameters are as follows: the exchange-correlation functional is the Perdew–Burke–Ernzerhof and generalized gradient approximation (PBE/GGA) method [40], the basis set is the double numerical plus polarization group (DNP) function [41], and the core treatment is the semi-core pseudopotentials (DSPPs) [38]. The convergence value of the total energy of the self-consistent field is 10^−6^ Ha, and the cut-off radius is set to 5.0 Å. Property calculations such as those for the binding energy, as well as the Raman and vibrational spectra, of the clusters are here performed using the PBE/GGA. The Raman spectra of the clusters are simulated by the wavelength of incident light at 514.5 nm and 10 K. Optical properties have been calculated using the time-dependent density functional theory [42] and PBE/GGA.

The binding energy is as follows:

(1) $$E_{b}=E_{\mathrm{total}}-n\times E_{A-\mathrm{atom}}-m\times E_{B-\mathrm{atom}}$$

where $E_{b}$ is the binding energy of the A-B system, $E_{\mathrm{total}}$ is the total energy of the A-B system, $E_{A-\mathrm{atom}}$ is the sum of atomic energies of the A atom, $E_{B-\mathrm{atom}}$ is the sum of atomic energies of the B atom, n is the number of the A atom, and m is the number of the B atom.

To a certain degree, the stability of the clusters can be determined by the second-order difference energy, which is defined as

(2) $$\Delta_{2}E=E(Ag_{n+1})+E(Ag_{n-1})-2E(Ag_{n})$$

(3) $$\Delta_{2}E=E(Ag_{n+1}Co_{1})+E(Ag_{n-1}Co_{1})-2E(Ag_{n}Co_{1})$$

where $E(Ag_{n})$ and $E(Ag_{n}\mathrm{Co})$ are the total energies of pure Ag and Ag–Co clusters, respectively.

According to Koopmans’ approximation, the electronegativity (*χ*), molecular softness (*S*), global hardness (*η*), and electrophilicity index (*ω*) play crucial roles as key indicators for nanoalloys, so we computed their *χ*, *S*, *η*, and *ω* using electron affinity (*EA*) and ionization energy (*IE*). The *χ*, *S*, *η*, *ω*, *IE*, and *EA* of all the nanoclusters are as follows:

(4) $$EA=-\varepsilon_{\mathrm{LUMO}}$$

(5) $$IE=-\varepsilon_{\mathrm{HOMO}}$$

(6) $$\chi =-\mu =\frac{IE+EA}{2}$$

(7) $$\eta =\frac{IE-EA}{2}$$

(8) $$S=\frac{1}{2\eta }$$

(9) $$\omega =S\mu^{2}$$

where *μ* is the chemical potential of the clusters.

## 3. Results and Discussion

**Figure 1:**
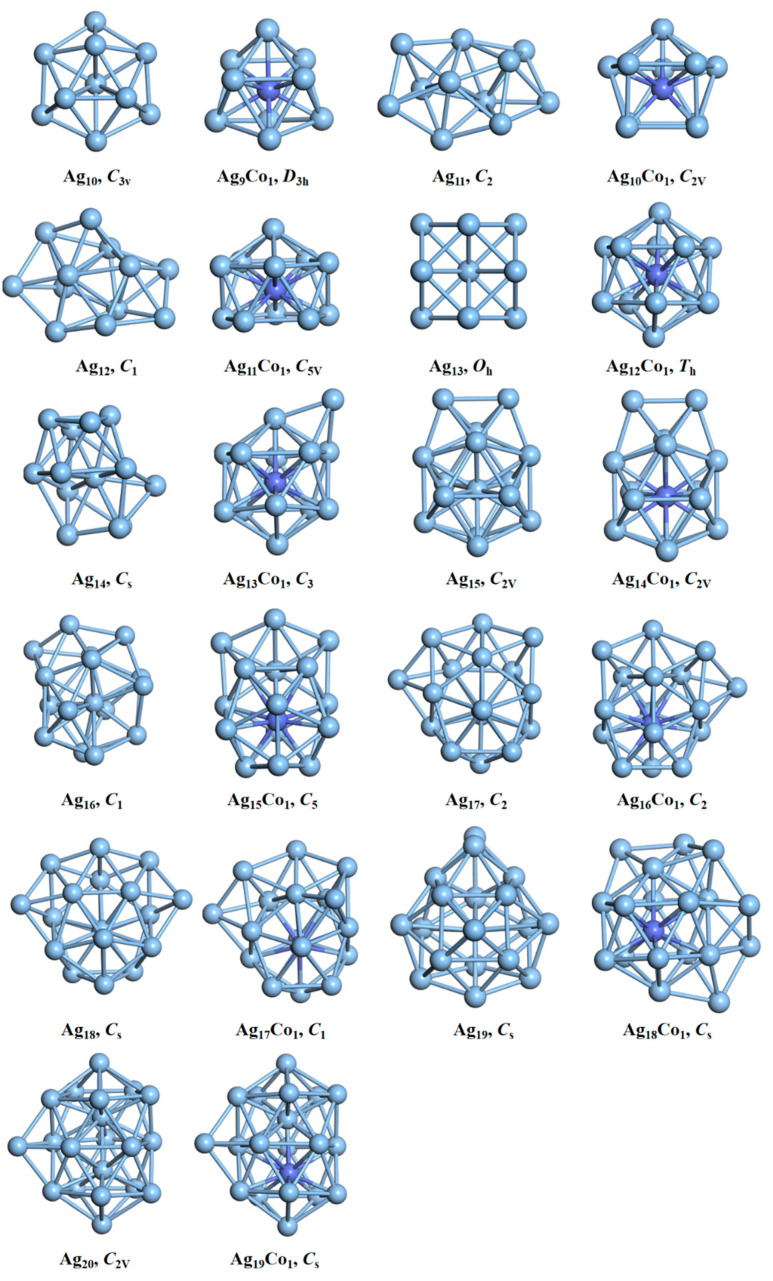
Structural images of the Ag–Co clusters. The cobalt atom is blue, and the point group symmetry is shown below the structure.

The schematic structures of the pure silver and silver–cobalt clusters after optimization are shown in Figure 1. The Ag_10_ cluster with *C*_3*v*_ point group symmetry is a hexahedral geometry, which is in agreement with the reported structure of the Ag_10_ cluster calculated using TPSS and M06 functionals [43]. When a single cobalt atom is doped, its lowest-energy structure does not maintain a hexahedral form, i.e., the Ag_9_Co_1_ cluster forms a core–shell structure with the Co atoms at the center and the remaining nine silver atoms at the edge. The Ag_11_ cluster adopts an amorphous structure connected by two decahedra, with *C*_2_ point group symmetry, which is in agreement with the previous report [44,45]. When one Co atom is doped, its lowest-energy structure displays a core–shell form exhibiting *C*_2*v*_ point group symmetry. The Ag_12_ cluster also has an amorphous structure, where all silver atoms lie on the outside, with low symmetry (*C*_1_). The Ag_11_Co_1_ cluster with *C*_5*v*_ point group symmetry shows a core–shell structure, where the Co atom is located in the central position. The lowest-energy structure of the Ag_13_ cluster, with a truncated octahedral shape maintaining the point group symmetry of *O_h_*, has been reported by our researchers [46,47]. While the lowest-energy structure of the Ag_12_Co_1_ cluster is an icosahedron core–shell form, the Co atom is in the center of icosahedral, and its point group symmetry is *T_h_*, which agrees with a previous report [32]. The Ag_14_ cluster with *C_s_* point group symmetry is deformed by adding a Ag atom to the truncated octahedral Ag_13_ cluster. The lowest-energy structure of the Ag_13_Co_1_ cluster entails the addition of an Ag atom on the icosahedral Ag_12_Co_1_ cluster, where the Co atom is also in the central position, and its point group symmetry is *C*_3_. The lowest-energy structure of the Ag_15_ cluster is icosahedral with the addition of two linked Ag atoms. The structure of the Ag_14_Co_1_ cluster is similar to that of the Ag_15_ cluster, and the Co atom is located in its center, and both have the point group symmetry of *C*_2*v*_. The structure of the Ag_16_ cluster is also based on an icosahedral form with the addition of three Ag atoms. Due to the addition of Ag atoms, the original icosahedral structure is slightly deformed, and its symmetry is reduced. The icosahedral structure with the addition of three Ag atoms in the Ag_15_Co_1_ cluster is not deformed, and also adopts a core–shell form. The Ag_17_ cluster is composed of an icosahedral shape with the addition of four Ag atoms, which is similar to previous reports [48]. The structure of the Ag_16_Co_1_ cluster closely resembles that of the Ag_17_ cluster, where the Co atom lies at its core. Both show the point group symmetry of *C*_2_. The Ag_18_ cluster has an icosahedral structure with the addition of five Ag atoms, which is in agreement with the previous result [43], and the Co atom of the Ag_17_Co_1_ cluster is also located in the core. The Ag_19_ cluster shows a deformed truncated octahedral structure with an additional six Ag atoms, which is also in agreement with the previous result [43], while the Ag_18_Co_1_ cluster has an icosahedral core–shell structure with the addition of six Ag atoms. The central position of the icosahedron is also occupied by the Co atom. The Ag_20_ cluster has an icosahedral structure with the addition of seven Ag atoms. The structure of the Ag_19_Co_1_ cluster is similar to that of the Ag_20_ cluster, and is a core–shell structure with a Co atom in the center. Generally speaking, when a single Co atom is doped into the silver cluster, the Ag–Co clusters can easily form core–shell structures, with Co atoms in the center, because the surface energy of the Co atom is larger than that of the Ag atom. As the number of atoms increases, the Ag clusters (except the Ag_13_ cluster) retain an almost icosahedral structure with the addition of some Ag atoms, and the Ag–Co cluster also includes some Ag atoms on top of the icosahedral structure. The electron configurations of Ag and Ag–Co clusters are shown in Table 2. There are two unpaired electrons in the Ag_10_, Ag_11_Co_1_, Ag_13_Co_1_, and Ag_19_Co_1_ clusters, so all are open-shell configurations, and their spin multiplicities are all 3. There are no unpaired electrons in the Ag_9_Co_1_, Ag_12_, Ag_14_, Ag_16_, Ag_15_Co_1_, Ag_18_, Ag_17_Co_1_, or Ag_20_ clusters, thus these are all closed-shell configurations, and their spin multiplicity is 1. There is only one unpaired electron in the Ag_11_, Ag_10_Co_1_, Ag_13_, Ag_17_, Ag_16_Co_1_, Ag_19_, and Ag_18_Co_1_ clusters, thus they are all open-shell configurations, and their spin multiplicities are all 2. There are three unpaired electron in the Ag_12_Co_1_, Ag_15_, and Ag_14_Co_1_ clusters, thus they all have open-shell configurations, and their spin multiplicities are 4.

**Figure 2:**
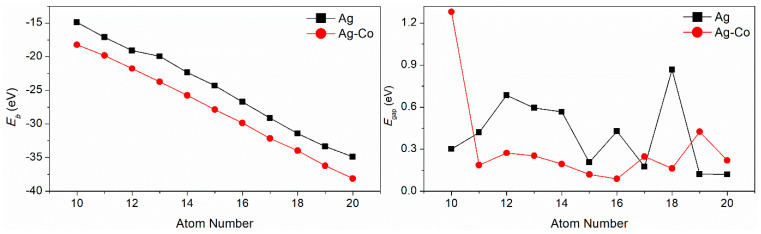
The *E*_b_ and *E*_gap_ values of the Ag–Co clusters.

The binding energy (*E*_b_) and the energy gap (*E*_gap_) between the energy of the highest occupied molecular orbital and the energy of the lowest unoccupied molecular orbital of the Ag–Co clusters are displayed in Figure 2. With an increase in the quantity of cluster atoms, their binding energy also escalates. When a single Co atom is doped into a silver cluster, its binding energy increases accordingly, because the cohesive energy and surface energy of Co atoms are greater than those of Ag atoms. For pure silver clusters, the energy gap values of Ag*_n_* (*n* = 10–14, 16, 18) clusters are larger than 0.3 eV, and the *E*_gap_ of the Ag_18_ cluster is the largest of all; the energy gap values of Ag*_n_* (*n* = 15, 17, 19, 20) clusters are lower than 0.3 eV, and the *E*_gap_ of the Ag_20_ cluster is lowest of all. The energy gap values of Ag*_n_*Co_1_ (*n* = 10–15, 17) clusters are lower than those of the corresponding pure silver clusters, and the energy gap values of Ag*_n_*Co_1_ (*n* = 9, 16, 18, 19) clusters are larger than those of the corresponding pure silver clusters. The *E*_gap_ of the Ag_9_Co_1_ cluster is the largest of all, and the *E*_gap_ of the Ag_15_Co_1_ cluster is the lowest of all. In a word, the energy gap of silver clusters can be tuned by the addition of a cobalt atom.

**Figure 3:**
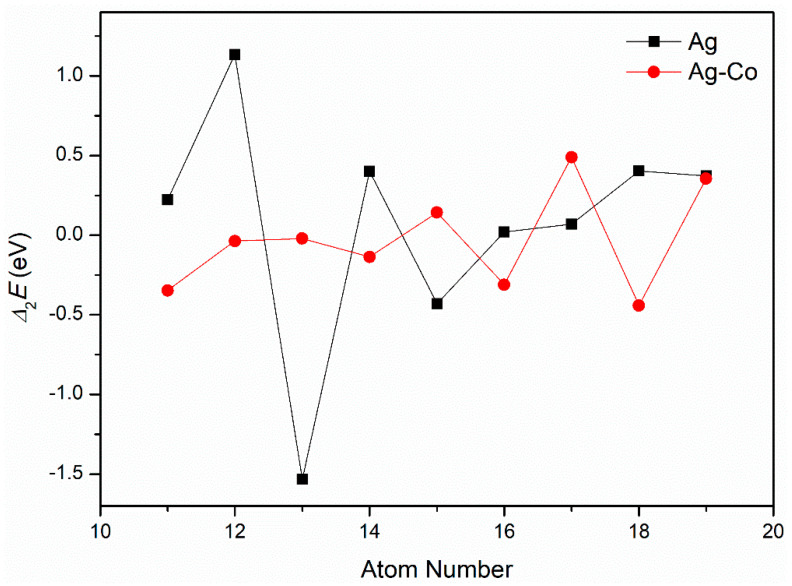
The Δ_2_*E* of the Ag–Co clusters.

The second-order differential energy values of pure Ag and Ag–Co clusters are given in Figure 3. For pure silver clusters, the $\Delta_{2}E$ values of the Ag_12_, Ag_14_, and Ag_18_ clusters are larger than those of their neighboring clusters, respectively; therefore, the Ag_12_, Ag_14_, and Ag_18_ clusters are relatively stable. For the Ag–Co clusters, the $\Delta_{2}E$ values of the Ag_12_Co_1_, Ag_14_Co_1_, and Ag_16_Co_1_ clusters are larger than those of their neighboring clusters, respectively; therefore, the stability of these clusters is relatively high.

**Figure 4:**
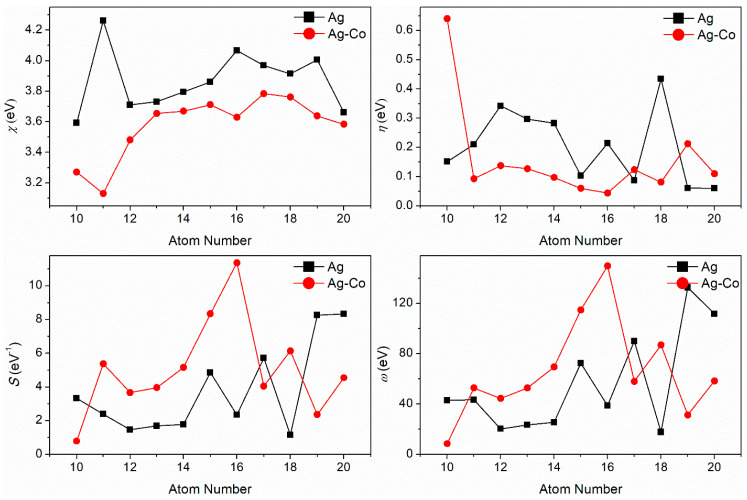
The *χ*, *η*, *S*, and *ω* of the Ag–Co clusters.

The *χ*, *S*, *η*, and *ω* of the pure Ag and Ag–Co clusters are shown in Figure 4. For pure Ag clusters, the electronegativity *χ* value increases with the number of atoms. The *χ* value of the Ag_11_ cluster is the highest, and the *χ* value of the Ag_10_ cluster is the lowest. These results indicate that the ability of the Ag_11_ cluster to obtain electrons is the strongest, and that of the Ag_10_ cluster is the weakest, the remaining clusters being in between the two. Comparing the *χ* values of pure silver clusters, we see that the *χ* value of the Ag–Co cluster decreases. For Ag–Co clusters, when the quantity of atoms reaches 10–13, the *χ* values firstly decrease and then increase; when the number of atoms is greater than 13, the *χ* values change very little, keeping around 3.7 eV. The *χ* value of the Ag_10_Co_1_ cluster is the lowest, and the *χ* value of the Ag_16_Co_1_ cluster is the highest. These results show that the ability of Ag–Co clusters to obtain electrons is reduced; that of the Ag_16_Co_1_ cluster is the strongest and that of the Ag_10_Co_1_ cluster is the weakest, and as the quantity of atoms increases, the ability of the Ag–Co clusters to obtain electrons firstly weakens, then increases, and then tends towards no change.

The trend in the global hardness *η* of pure Ag clusters and Ag–Co clusters is consistent with that of the energy gap between the energy of the highest occupied molecular orbital and the energy of the lowest unoccupied molecular orbital. For pure silver clusters, the *η* value of the Ag_18_ cluster is the highest, and the *η* value of the Ag_20_ cluster is the lowest. For Ag–Co clusters, the *η* value of the Ag_9_Co_1_ cluster is the highest, and the *η* value of the Ag_15_Co_1_ cluster is the lowest. These results illustrate that the electronic stability of the Ag_18_ and Ag_9_Co_1_ clusters is high, and that of the Ag_20_ and Ag_15_Co_1_ clusters is low.

The change trends of the molecular softness *S* of the Ag and Ag–Co clusters are the opposite to those of their global hardness. For pure Ag clusters, the *S* values of the Ag_10_, Ag_15_, Ag_17_, Ag_19_, and Ag_20_ clusters are greater than 3.0 eV^−1^, and those of the remaining pure Ag clusters are lower than 3.0 eV^−1^. The *S* value of the Ag_20_ cluster is the highest, and the *S* value of the Ag_18_ cluster is the lowest. The *S* values of the Ag*_n_*Co_1_ (*n* = 9–15, 17) clusters are increased with the doping of the Co atom, and the *S* values of the Ag*_n_*Co_1_ (*n* = 9, 16, 18, 19) clusters decrease with the doping of the Co atom. The *S* value of the Ag_15_Co_1_ cluster is the highest, and the *S* value of the Ag_9_Co_1_ cluster is the lowest.

Overall, the change trends in the electrophilicity index *ω* of the Ag (except Ag_11_ and Ag_20_) and Ag–Co clusters are consistent with their molecular softness. The *ω* of the Ag_11_ cluster is greater than that of the Ag_10_ cluster, which shows a different trend from their *S* change trend. At the same time, the *ω* of the Ag_20_ cluster is lower than that of the Ag_19_ cluster, which is also different from their *S* change trend. These results illustrate that the *ω* values of the Ag (except Ag_11_ and Ag_20_) and Ag–Co clusters are mainly determined by their molecular softness, while the *ω* values of the Ag_11_ and Ag_20_ clusters are primarily influenced by their electronegativity. The *ω* value of the Ag_19_ cluster is the highest, and the *ω* value of the Ag_18_ cluster is the lowest. The *ω* value of the Ag_16_Co_1_ cluster is the highest, and the *ω* value of the Ag_9_Co_1_ cluster is the lowest.

**Figure 5:**
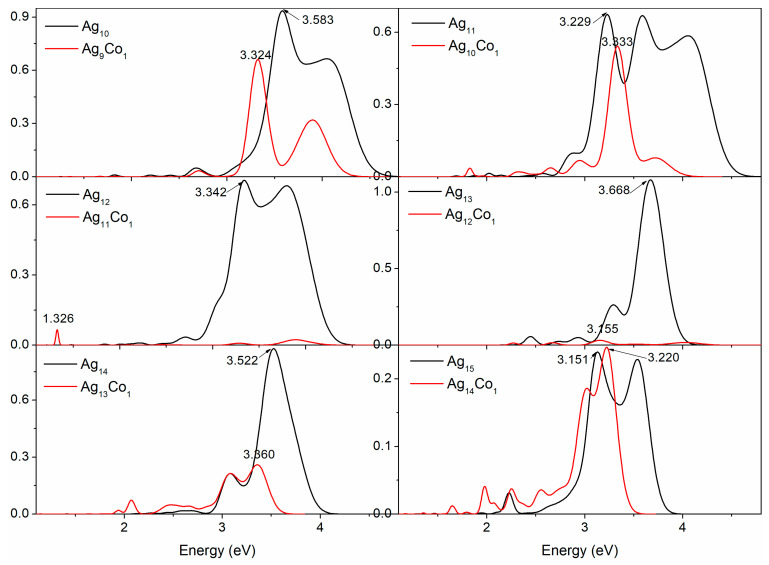
The optical spectra of the 10–15-atom Ag–Co clusters.

In the field of materials science, the spectral analysis of metal clusters is a crucial research approach. Through further study, we obtained spectra for the 10–15-atom Ag–Co clusters (Figure 5) and analyzed them in detail. These spectra present a sequence of distinct absorption peaks, and the appearance of these peaks indicates excited electrons in these clusters, revealing for us the electronic-level structure of the clusters. The spectrum of the Ag_10_ cluster presents three significant absorption peaks, of which the peak at 4.052 eV is close to the experimental peak at 3.98 eV [44] or 3.97 eV [49], and the strongest peak is located at 3.583 eV. When a Co atom is doped into the Ag_10_ cluster forming the Ag_9_Co_1_ cluster, the spectrum of the Ag_9_Co_1_ cluster also shows three distinct absorption peaks, and the strongest peak of this cluster is located at 3.324 eV. Consequently, a spectral redshift is observed in the Ag_9_Co_1_ cluster in comparison to the Ag_10_ cluster. The spectrum of the Ag_11_ cluster shows three significantly strong absorption peaks and three small peaks, which can be seen in the experimental spectrum of the Ag_11_ cluster [44,50,51]; the strongest peak is located at 3.229 eV. The spectrum of the Ag_10_Co_1_ cluster also displays one strong peak and five small peaks, and the strongest peak of this cluster is located at 3.333 eV. The spectrum of the Ag_10_Co_1_ cluster exhibits a blueshift of 0.104 eV in comparison to that of the Ag_11_ cluster. There are two strong peaks and two small peaks in the spectrum of the Ag_12_ cluster, which are in agreement with the experimental peaks [44]; the strongest peak is located at 3.342 eV. When the Co atom is in the Ag_12_ cluster, the absorption peaks become too weak; there are three small absorption peaks, and the strongest peak is located at 1.326 eV. Compared with the spectrum of the Ag_12_ cluster, a big redshift in the spectrum of the Ag_11_Co_1_ cluster is observed. There is one strong peak and four small peaks in the spectrum of the truncated octahedral Ag_13_ cluster, which findings are in agreement with experimental peaks [44] and our previous findings [46], wherein the strongest peak lies at 3.668 eV. While the peaks of the optical spectrum of the icosahedral Ag_12_Co_1_ cluster become very weak, the strongest peak lies at 3.155 eV. A redshift in the spectrum of the Ag_12_Co_1_ cluster is observed compared with the Ag_13_ cluster. There is one strong peak and three small peaks in the spectrum of the Ag_14_ cluster; the strongest peak lies at 3.522 eV, which is close to the experimental peak at 3.48 eV [44]. With a Co atom doped in the Ag_14_ cluster, the spectrum of the Ag_13_Co_1_ cluster has more peaks than that of the Ag_14_ cluster and shows six peaks, the strongest peak lying at 3.360 eV. The spectrum of the Ag_13_Co_1_ cluster exhibits a redshift of 0.162 eV in comparison to that of the Ag_14_ cluster. There are two strong peaks and one small peak in the spectrum of the Ag_15_ cluster, the strongest peak lying at 3.131 eV, and a peak at 3.532 eV is observed in the experimental spectrum [44]. At the same time, with a Co atom doped in the Ag_15_ cluster, the spectrum of the Ag_14_Co_1_ cluster has more peaks than that of the Ag_15_ cluster, and shows two strong peaks and five small peaks, the strongest peak lying at 3.220 eV. There is a 0.089 eV blueshift observed in the spectrum of the Ag_14_Co_1_ cluster in comparison to the Ag_15_ cluster.

**Figure 6:**
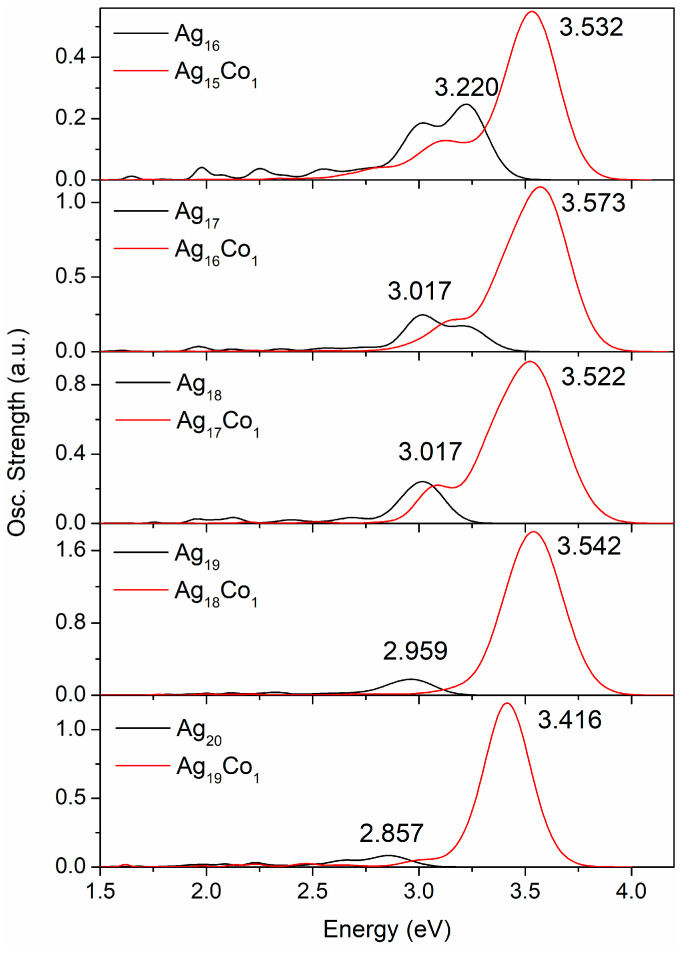
The optical spectra of the 16–20-atom Ag–Co clusters.

When the number of atoms in the Ag–Co clusters is changed from 16 to 20, their spectra show some remarkable change patterns. Therefore, the spectra of the 16–20-atom Ag–Co clusters are drawn separately, as shown in Figure 6. There are two strong peaks and five small peaks in the spectrum of the Ag_16_ cluster, the strongest peak lying at 3.220 eV. There are only two peaks in the spectrum of the Ag_15_Co_1_ cluster, the strongest peak lying at 3.532 eV. Two strong peaks and four small peaks are observed in the spectrum of the Ag_17_ cluster, the strongest peak lying at 3.017 eV, and the other peak lies at 3.123 eV. Only one peak is observed in the spectrum of the Ag_16_Co_1_ cluster and this lies at 3.573 eV. There is one strong peak and four small peaks in the spectrum of the Ag_18_ cluster, with the strongest peak also lying at 3.017 eV. There are two peaks in the spectrum of the Ag_17_Co_1_ cluster, with the strongest peak lying at 3.522 eV. The spectrum of the Ag_19_ cluster exhibits one clear peak and three small peaks, with the strongest peak lying at 2.959 eV. The spectrum of the Ag_18_Co_1_ cluster presents only one peak, which lies at 3.542 eV. The spectrum of the Ag_20_ cluster displays one obvious peak and four small peaks, and the strong peak lies at 2.857 eV. The spectrum of the Ag_19_Co_1_ cluster shows only one strong peak and three small peaks, with the strongest peak at 3.416 eV. Thus, the addition of a Co atom into Ag clusters causes a strong peak at approximately 3.5 eV in the spectra of the Ag–Co clusters, and a blueshift in the spectra of the Ag–Co clusters compared with the same number of atoms in a pure silver cluster.

**Figure 7:**
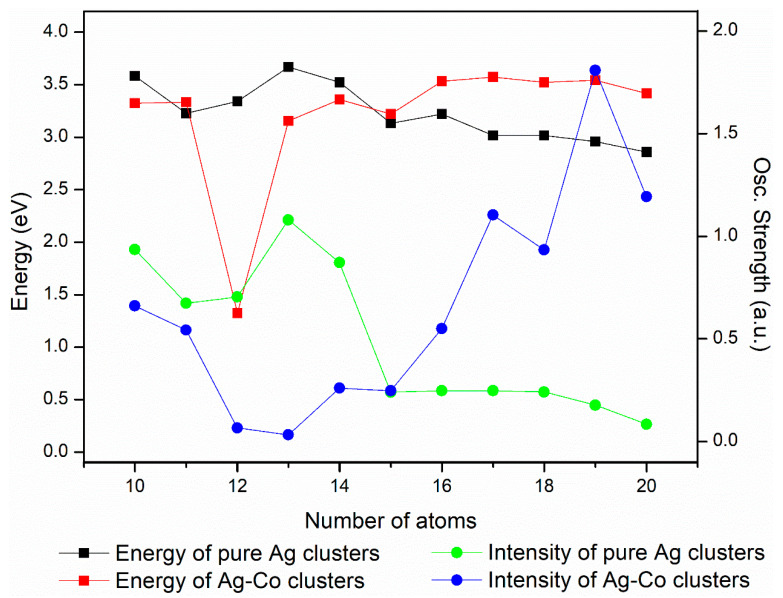
Energy and intensity of optical peaks of Ag–Co clusters vs. the number of atoms.

Generally speaking, the spectral analysis of the Ag–Co clusters has provided valuable insights into their electronic structure and the influence of introducing Co into these clusters on their optical properties. Figure 7 displays the energy and intensity of the optical peaks of the Ag–Co clusters with increasing numbers of atoms. For the pure Ag clusters, the energy and intensity of their spectra generally change little as the number of atoms increases, i.e., redshift occurs as the number of atoms grows. For the Ag–Co clusters, the energy of their spectra overall exhibits little change as the number of atoms grows; the strongest peaks are roughly distributed at 3.5 eV, and the intensity of their spectra strengthens overall. When the numbers of atoms are 10, 12, 13, and 14, the addition of the Co atom makes the optical spectra redshift and weakens the intensity. As the number of atoms is 15–20, doping with Co leads to a redshift of the optical spectra and a strengthening of intensity. The blueshift or redshift in the absorption peaks with the addition of a Co atom suggests that the Co atom has the ability to adjust the electronic characteristics of the Ag–Co clusters efficiently. These findings have important implications for the development of novel optical materials and the understanding of the fundamental processes governing the electronic properties of metal clusters.

**Figure 8:**
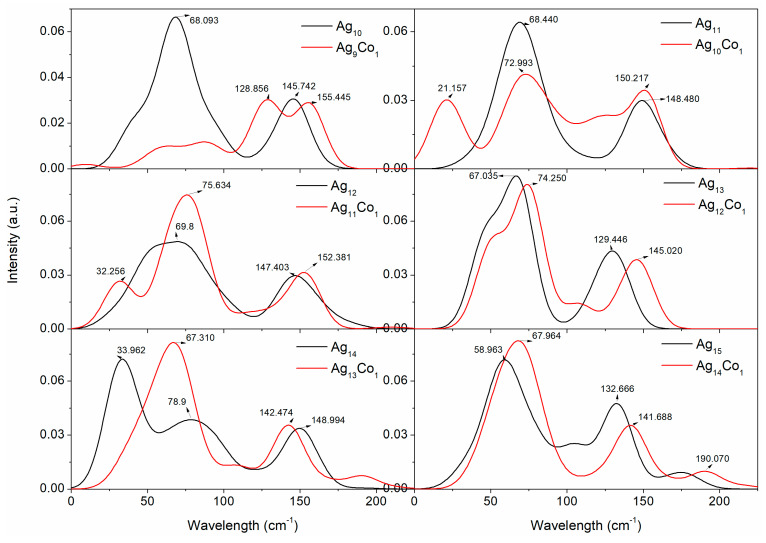
The Raman spectra of the 10–15-atom Ag–Co clusters.

**Figure 9:**
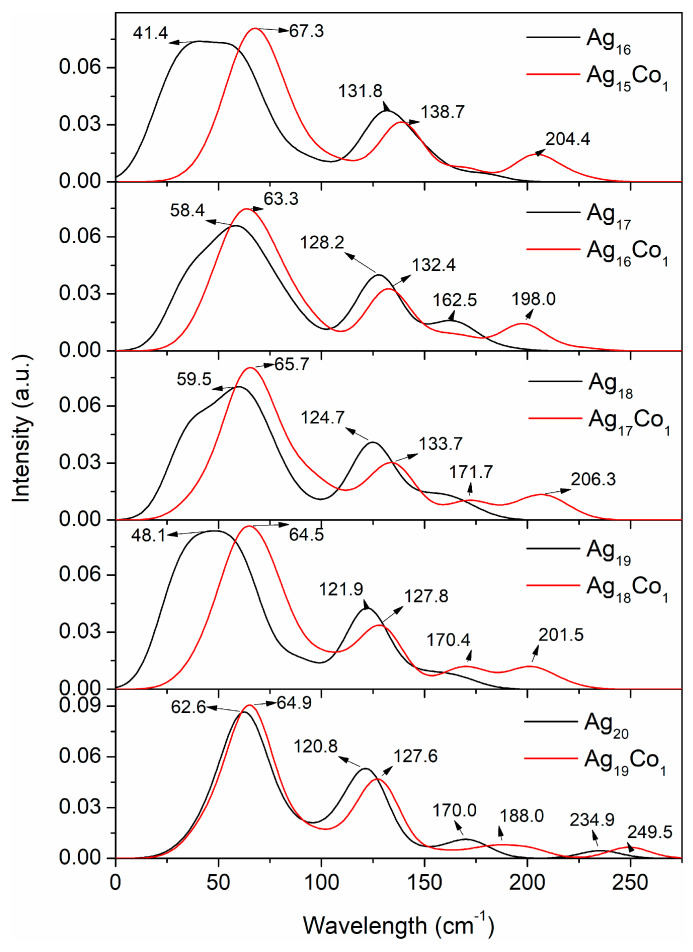
The Raman spectra of the 16–20-atom Ag–Co clusters.

Raman spectroscopy plays a crucial role in studying the structure, chemical composition, size effects, and magnetic properties of Ag–Co clusters. By analyzing the Raman spectra of Ag–Co clusters, we can gain an insight into their basic properties, and lay the foundation for subsequent experimental and applied studies. Thus, the Raman spectra of the Ag–Co clusters were investigated by GGA/PBE, and the Raman spectra are displayed in Figure 8 and Figure 9. The range in the Raman spectra of the 10–15-atom Ag–Co clusters is small, and the range in the Raman spectra of the 16–20-atom Ag–Co clusters is large; thus, Figure 8 and Figure 9 show the Raman spectra of the 10–15-atom and 16–20-atom Ag–Co clusters, respectively. The Raman spectrum of the Ag_10_ cluster presents two peaks at 68.1 and 145.7 cm^−1^, respectively (Figure 8). The addition of a Co atom into the Ag_10_ cluster leads to the appearance of five peaks, lying at 9.2, 63.4, 86.9, 128.9, and 155.4 cm^−1^, respectively (Figure 8). The Raman spectrum of the Ag_11_ cluster also exhibits two peaks, located at 68.4 and 148.5 cm^−1^, respectively (Figure 8). The presence of one Co atom in the Ag_11_ cluster leads to the appearance of two new Raman peaks at 21.2 and 125.9 cm^−1^ and increases the intensity of the Raman peaks at 150.2 cm^−1^, while it weakens the intensity of the Raman peaks at 73.0 cm^−1^ (Figure 8). The Raman spectrum of the Ag_12_ cluster also exhibits two peaks, with center at 69.8 and 147.4 cm^−1^ (Figure 8). The appearance of a new Raman peak at 32.3 cm^−1^ and the increasing intensity of the Raman peak at 75.6 cm^−1^ are attributed to the cooperative effect of the Co atom in the cluster (Figure 8). The Raman spectrum of the Ag_13_ cluster also displays two peaks, with centers at 67.0 and 129.4 cm^−1^ (Figure 8), which findings are in agreement with our previous results [46]. The presence of one Co atom in the Ag_13_ cluster leads to the appearance of a new Raman peak at 106.7 cm^−1^, and the Raman peaks at 74.3 and 145.0 cm^−1^ shift to a larger wavelength (Figure 8). The Raman spectrum of the Ag_14_ cluster displays three distinctive Raman peaks, which lie at 34.0, 78.9, and 149 cm^−1^ (Figure 8). The Raman spectrum of the Ag_13_Co_1_ cluster displays four distinct Raman peaks, which lie at 67.3, 106.6, 142.5, and 189.6 cm^−1^ (Figure 8). The Raman spectrum of the Ag_15_ cluster has four distinct peaks, centered at 59.0, 104.3, 132.7, and 174.6 cm^−1^ (Figure 8). With the addition of a Co atom into the Ag_15_ cluster, the three Raman peaks at 68.0, 141.7, and 19.1 cm^−1^ shift to a larger wavelength in comparison to the Raman spectrum obtained from the Ag_15_ cluster (Figure 8).

The Raman spectrum of the Ag_16_ cluster exhibits two Raman peaks, with centers at 41.4 and 131.8 cm^−1^ (Figure 9). When a Co atom is doped into the Ag_16_ cluster, the Raman spectrum shows a new peak at 204.4 cm^−1^, and the other two peaks shift to greater wavelengths of 67.3 and 138.7 cm^−1^ (Figure 9). Both Raman spectra of the Ag_17_ and Ag_16_Co_1_ clusters present three Raman peaks; all peaks of the Ag_16_Co_1_ clusters shift towards larger wavelengths compared with the Ag_17_ cluster (Figure 9). There are two Raman peaks at 59.5 and 124.7 cm^−1^ in the Raman spectrum of the Ag_18_ cluster (Figure 9). In contrast to the Raman spectrum of the Ag_18_ cluster, two new Raman peaks appear at 171.7 and 206.3 cm^−1^, and all Raman peaks shift to larger wavelengths (Figure 9). The Raman spectra of the 19-atom Ag–Co clusters are similar to those of the 18-atom Ag–Co clusters; the Raman spectrum of the Ag_18_ cluster shows two Raman peaks at 48.1 and 121.9 cm^−1^, and the Raman spectrum of the Ag_18_Co_1_ cluster shows four Raman peaks at 64.5, 127.8, 170.4, and 201.5 cm^−1^ (Figure 9). Both Raman spectra of the 20-atom Ag–Co cluster display four Raman peaks, and all Raman peaks of the Ag_19_Co_1_ cluster shift to the large wavelength area compared with the Ag_20_ cluster (Figure 9). These findings suggest that the Raman spectra of the Ag–Co clusters can be used to monitor the structural changes in the clusters as the number of Co atoms increases.

**Figure 10:**
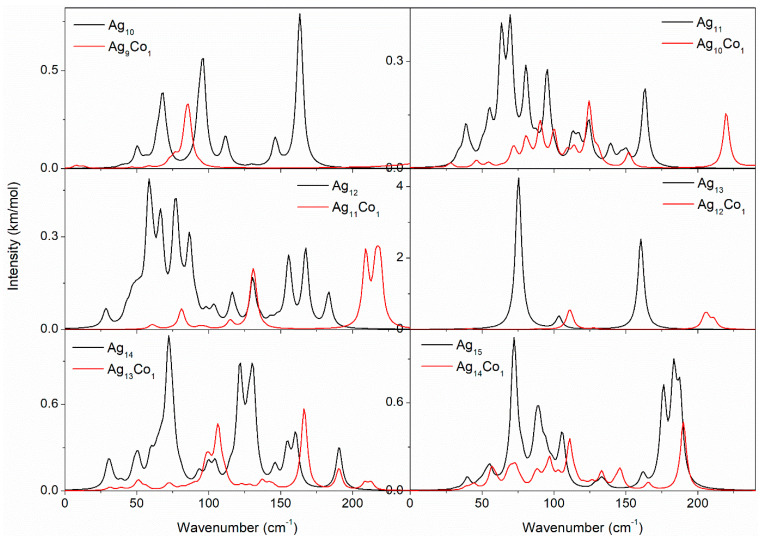
The vibrational spectra of the 10–15-atom Ag–Co clusters.

As an effective characterization method, the vibrational spectrum is important for revealing the structure, performance, and stability of Ag–Co clusters. Thus the vibrational spectra of the Ag–Co clusters are studied by GGA/PBE. The vibrational spectra of the 10–15-atom Ag–Co clusters are displayed in Figure 10. The vibrational spectrum of the Ag_10_ cluster exhibits six peaks at 50.3, 68.3, 96.3, 111.3, 146.3, and 163.3 cm^−1^, while the vibrational spectrum of the Ag_9_Co_1_ cluster exhibits only one significant peak at 85.7 cm^−1^. This may indicate that the vibrational pattern is more complex in the Ag_10_ cluster and relatively simple in the Ag_9_Co_1_ cluster. Both vibrational spectra of the Ag_11_ and Ag_10_Co_1_ clusters are more complex; the Ag_10_Co_1_ cluster has a strong vibrational peak at 262.5 cm^−1^, while the vibrational peak of the Ag_11_ cluster is located below a wavenumber of 175.0 cm^−1^. The vibrational spectrum of the Ag_12_ cluster exhibits thirteen peaks, while the vibrational spectrum of the Ag_11_Co_1_ cluster shows six peaks, suggesting the vibrational pattern of the Ag_11_Co_1_ cluster is simpler than that of the Ag_12_ cluster. Both vibrational spectra of the Ag_13_ and Ag_12_Co_1_ clusters exhibit only three peaks; the vibrational peaks of the Ag_13_ cluster are located at 75.5, 103.5, and 160.5 cm^−1^, which is in agreement with our previous results [46], while the vibrational peaks of the Ag_12_Co_1_ cluster locate at 111.4, 205.4, and 210.4 cm^−1^. The vibrational spectrum of the Ag_14_ cluster appears to be more complex, while the vibrational spectrum of the Ag_13_Co_1_ cluster displays a simple vibrational pattern, indicating that the addition of the Co atom leads to a simplifying of the vibrational pattern. Both vibrational spectra of the Ag_15_ and Ag_14_Co_1_ clusters are more complex, and their vibrational peaks are in almost the same positions. In the Ag_14_Co_1_ cluster, a vibrational peak is observed at 145.9 cm^−1^, but no vibrational peaks at 176.3 and 183.3 cm^−1^ are observed.

**Figure 11:**
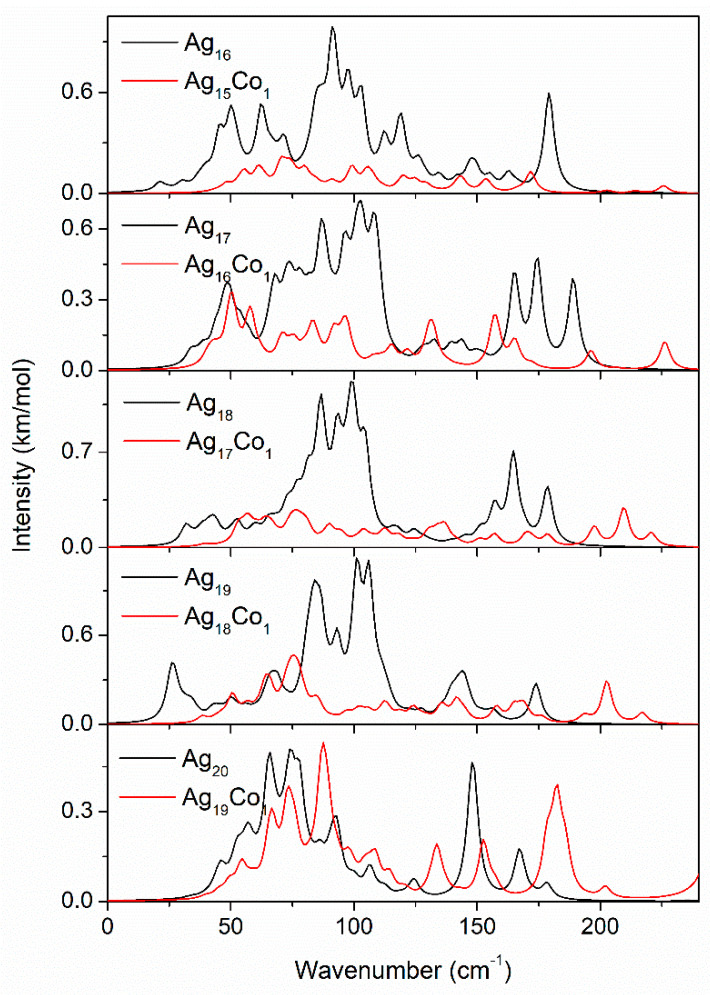
The vibrational spectra of the 16–20-atom Ag–Co clusters.

The vibrational spectra of the 16–20-atom Ag–Co clusters are displayed in Figure 11. Both the vibrational spectra of the Ag_16_ and Ag_15_Co_1_ clusters are more complex, and the intensity of the vibrational peaks of the Ag_16_ cluster is stronger than that of the Ag_15_Co_1_ cluster. A new vibrational peak at 225.7 cm^−1^ is observed in the Ag_15_Co_1_ cluster. The vibrational spectra of the Ag_17_ and Ag_16_Co_1_ clusters are similar to those of the Ag_16_ and Ag_15_Co_1_ clusters; they are more complex, and a new vibrational peak at 225.8 cm^−1^ is observed in the Ag_16_Co_1_ cluster. The vibrational spectra of the 18-, 19- and 20-atom Ag and Ag–Co clusters are also similar to those of the Ag_16_ and Ag_15_Co_1_ clusters, and they are also more complex; however, the vibrational spectra of the 18-, 19-, and 20-atom Ag–Co clusters exhibit some new vibrational peaks above 200 cm^−1^. These findings can serve as a basis for further investigations into the Ag–Co clusters and their potential applications in various fields. Moreover, the vibrational spectra presented here can serve as a reference for the characterization of other Ag–Co clusters and related systems, contributing to a deeper understanding of their structural and dynamic properties.

**Figure 12:**
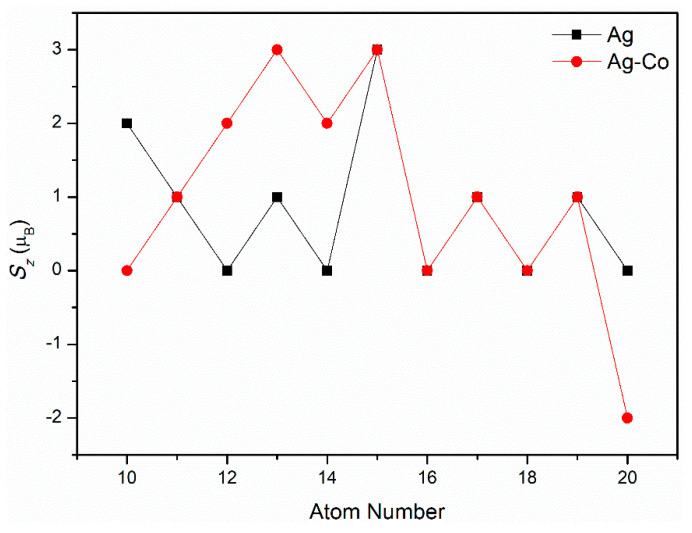
The magnetic properties of Ag–Co clusters.

The magnetic characteristics of the Ag–Co clusters are displayed in Figure 12. As is evident from Figure 12, the magnetic characteristics of the Ag–Co clusters are quite intriguing. The magnetic moment of the Ag_10_ cluster is 2 µ_B_, which is different from those of the Ag_12_, Ag_14_, Ag_16_, Ag_18_, and Ag_20_ clusters, for all of which the magnetic moments are zero. The magnetic moment of the Ag_15_ cluster is 3 µ_B_, which is different from those of the Ag_11_, Ag_13_, Ag_17_, and Ag_19_ clusters, for all of which the magnetic moments are 1 µ_B_. The addition of the Co atom leads to the magnetic moments of the Ag_11_Co_1_, Ag_13_Co_1_, and Ag_19_Co_1_ clusters increasing to 2 µ_B_, and that of the Ag_12_Co_1_ cluster increasing to 3 µ_B_, while that of the Ag_9_Co_1_ cluster decreases to zero. The magnetic moments of the Ag_10_Co_1_, Ag_14_Co_1_, Ag_15_Co_1_, Ag_16_Co_1_, Ag_17_Co_1_, and Ag_18_Co_1_ clusters, on the other hand, remain unchanged—both the magnetic moments of the Ag_15_Co_1_ and Ag_17_Co_1_ clusters are zero, all the magnetic moments of the Ag_10_Co_1_, Ag_16_Co_1_, and Ag_18_Co_1_ clusters are 1 µ_B_, and the magnetic moment of the Ag_14_Co_1_ cluster is 3 µ_B_. The current study serves as a starting point for researchers interested in exploring the fascinating world of Ag–Co clusters and their unique magnetic properties. Future investigations are expected to reveal more about the intricate interactions between Ag and Co atoms, opening up new avenues for the development of advanced magnetic materials.

## 4. Conclusions

The structural, electronic, magnetic, and optical characteristics of Co-doped 10–20-atom silver clusters were investigated by GGA/PBE. The Ag–Co clusters easily form core–shell structures, with Co atom in the center. With a growth in the quantity of atoms, the Ag clusters (except the Ag_13_ cluster) retain an almost icosahedral structure, with the addition of some Ag atoms, and Ag–Co clusters also receive some Ag atoms on top of their icosahedral structure. The energy gap values of Ag*_n_*Co_1_ (*n* = 10–15, 17) clusters are lower than the ones for pure silver clusters; the energy gap values of Ag*_n_*Co_1_ (*n* = 9, 16, 18, 19) clusters are larger than those of pure silver clusters. The electronegativity *χ* value of the Ag_16_Co_1_ cluster is the strongest. The *S* values of the Ag*_n_*Co_1_ (*n* = 9–15, 17) clusters increase when doping the Co atom, and the *S* values of the Ag*_n_*Co_1_ (*n* = 9, 16, 18, 19) clusters decrease when doping the Co atom. The *S* value of the Ag_15_Co_1_ cluster is the greatest. The *ω* value of the Ag_16_Co_1_ cluster is greatest. In the optical spectra of pure Ag clusters, redshift occurs with an increasing number of atoms. In the optical spectra of Ag–Co clusters, the energy of their spectra overall exhibits little change with an increasing number of atoms; the strongest peaks are roughly distributed around 3.5 eV, and the overall intensity of their spectra strengthens. The Raman and vibrational spectra of the Ag–Co clusters can be used to monitor structural changes in the clusters as the number of Co atoms increases. The addition of a Co atom leads to the magnetic moments of the Ag_11_Co_1_, Ag_13_Co_1_, and Ag_19_Co_1_ clusters increasing to 2 µ_B_, that of the Ag_12_Co_1_ cluster increasing to 3 µ_B_, and that of the Ag_9_Co_1_ cluster decreasing to zero, while the magnetic moments of the Ag_10_Co_1_, Ag_14_Co_1_, Ag_15_Co_1_, Ag_16_Co_1_, Ag_17_Co_1_, and Ag_18_Co_1_ clusters remain unchanged. Therefore, this study on the performances of Ag–Co double metal clusters could provide important guidance for their application in optical and photoelectric devices.

## Figures and Tables

**Table 1:** Gupta potential parameters for Ag–Ag, Ag–Co, and Co–Co interactions [36,37].

	*A* (eV)	ξ (eV)	*p*	*q*	*r*_0_ (Å)
Ag–Ag	0.1043	1.1940	2.8900	10.7900	3.1900
Ag–Co	0.1444	1.4776	2.6950	10.0100	3.0850
Co–Co	0.1757	1.8430	2.5000	9.2100	2.7950

**Table 2:** The electron configurations of Ag and Ag–Co clusters.

	Unpaired Electrons	Closed- or Open-Shell	Spin Multiplicity
Ag_10_	2	open-shell	3
Ag_9_Co_1_	0	closed-shell	1
Ag_11_	1	open-shell	2
Ag_10_Co_1_	1	open-shell	2
Ag_12_	0	closed-shell	1
Ag_11_Co_1_	2	open-shell	3
Ag_13_	1	open-shell	2
Ag_12_Co_1_	3	open-shell	4
Ag_14_	0	closed-shell	1
Ag_13_Co_1_	2	open-shell	3
Ag_15_	3	open-shell	4
Ag_14_Co_1_	3	open-shell	4
Ag_16_	0	closed-shell	1
Ag_15_Co_1_	0	closed-shell	1
Ag_17_	1	open-shell	2
Ag_16_Co_1_	1	open-shell	2
Ag_18_	0	closed-shell	1
Ag_17_Co_1_	0	closed-shell	1
Ag_19_	1	open-shell	2
Ag_18_Co_1_	1	open-shell	2
Ag_20_	0	closed-shell	1
Ag_19_Co_1_	2	open-shell	3

## Data Availability

The authors declare that all data supporting the findings of this study are available within the article.
